# END-OF-LIFE SERVICES IN TRIBAL COMMUNITIES

**DOI:** 10.1093/geroni/igz038.2469

**Published:** 2019-11-08

**Authors:** Lori L Jervis, Derrell W Cox

**Affiliations:** University of OKlahoma, Norman, Oklahoma, United States

## Abstract

Terminally ill American Indians/Alaska Natives (AI/ANs) are less likely to receive hospice and palliative care than other racial/ethnic groups, with fewer than 1/3 receiving these services compared to over 45% of EuroAmericans (Johnson, 2013; NHPCO, 2017). While some AI/ANs believe that End of Life (EoL) services will hasten their deaths (Colclough & Brown, 2014), claims that Natives reject EoL services due to death taboos are likely overgeneralizations. Rather, extant studies point to barriers to access resulting from lack of financial resources and inadequate service infrastructure, especially in rural areas (Jervis, Jackson, & Manson, 2002; Kitzes & Berger, 2004; Kitzes & Domer, 2004; Weech-Maldonado et al., 2003). While these factors undoubtedly play a role in underutilization, our preliminary research suggests that other factors—such as a lack of tribally based EoL programs and the cultural mismatches that occur when non-Native programs attempt to deliver hospice services to Native clients—may discourage AIANs from seeking and/or retaining these services. In this presentation, we report on results from a nationwide telephone survey of the availability of EoL care across AIAN tribes. We also present findings from in-depth interviews with local service providers on the challenges and successes they experienced in providing EoL care to their AI clients in one tribal community. Together, these findings will add to our growing understanding of the factors that inhibit and facilitate EoL service utilization, and suggest possibilities for improving access.

